# Supplementary material for: Time-series transcriptome analysis identified differentially expressed genes in broiler chicken infected with mixed Eimeria species
Source: Front Genet. 2022 Aug 8;13:886781. doi: 10.3389/fgene.2022.886781 (PMC9393255; doi:10.3389/fgene.2022.886781)
Supplement: Supplementary file 2 [file DataSheet1.ZIP › 7dpi_GO.Gsea.1625106495923/GOCC_INNER_MITOCHONDRIAL_MEMBRANE_PROTEIN_COMPLEX.html]

Details for gene set GOCC\_INNER\_MITOCHONDRIAL\_MEMBRANE\_PROTEIN\_COMPLEX[GSEA]

|  || Dataset | TMM\_7dpi\_gct\_format\_7dpi\_gct\_format.Class\_4dpi.cls #PC\_versus\_NC.Class\_4dpi.cls #PC\_versus\_NC\_repos |
| Phenotype | Class\_4dpi.cls#PC\_versus\_NC\_repos |
| Upregulated in class | 1 |
| GeneSet | GOCC\_INNER\_MITOCHONDRIAL\_MEMBRANE\_PROTEIN\_COMPLEX |
| Enrichment Score (ES) | 0.705554 |
| Normalized Enrichment Score (NES) | 3.5063093 |
| Nominal p-value | 0.0 |
| FDR q-value | 0.0 |
| FWER p-Value | 0.0 |
Table: GSEA Results Summary

  

Fig 1: Enrichment plot: GOCC\_INNER\_MITOCHONDRIAL\_MEMBRANE\_PROTEIN\_COMPLEX      
 Profile of the Running ES Score & Positions of GeneSet Members on the Rank Ordered List

  

| SYMBOL | TITLE | RANK IN GENE LIST | RANK METRIC SCORE | RUNNING ES | CORE ENRICHMENT || 1 | TIMM10B | na | 146 | 1.149 | 0.0151 | Yes |
| 2 | DNAJC19 | na | 208 | 1.036 | 0.0347 | Yes |
| 3 | COX5A | na | 300 | 0.942 | 0.0495 | Yes |
| 4 | NDUFA2 | na | 303 | 0.935 | 0.0716 | Yes |
| 5 | NDUFB6 | na | 307 | 0.933 | 0.0936 | Yes |
| 6 | NDUFS5 | na | 308 | 0.932 | 0.1158 | Yes |
| 7 | CHCHD10 | na | 312 | 0.929 | 0.1377 | Yes |
| 8 | NDUFB7 | na | 323 | 0.915 | 0.1587 | Yes |
| 9 | NDUFB2 | na | 348 | 0.885 | 0.1778 | Yes |
| 10 | NDUFAB1 | na | 356 | 0.876 | 0.1981 | Yes |
| 11 | NDUFA7 | na | 371 | 0.857 | 0.2173 | Yes |
| 12 | NDUFC2 | na | 394 | 0.834 | 0.2353 | Yes |
| 13 | UQCR10 | na | 444 | 0.785 | 0.2499 | Yes |
| 14 | COX6A1 | na | 480 | 0.760 | 0.2651 | Yes |
| 15 | UQCRB | na | 487 | 0.753 | 0.2825 | Yes |
| 16 | NDUFA11 | na | 497 | 0.746 | 0.2996 | Yes |
| 17 | SDHAF4 | na | 516 | 0.730 | 0.3154 | Yes |
| 18 | SMDT1 | na | 548 | 0.711 | 0.3298 | Yes |
| 19 | NDUFA13 | na | 557 | 0.708 | 0.3460 | Yes |
| 20 | NDUFS3 | na | 623 | 0.675 | 0.3566 | Yes |
| 21 | NDUFB3 | na | 656 | 0.657 | 0.3696 | Yes |
| 22 | NDUFS8 | na | 661 | 0.653 | 0.3848 | Yes |
| 23 | NDUFS6 | na | 686 | 0.642 | 0.3981 | Yes |
| 24 | SDHD | na | 708 | 0.632 | 0.4114 | Yes |
| 25 | UQCC3 | na | 735 | 0.620 | 0.4239 | Yes |
| 26 | NDUFA1 | na | 742 | 0.616 | 0.4381 | Yes |
| 27 | UQCRHL | na | 788 | 0.594 | 0.4485 | Yes |
| 28 | NDUFA5 | na | 794 | 0.593 | 0.4622 | Yes |
| 29 | SDHB | na | 840 | 0.575 | 0.4722 | Yes |
| 30 | DNAJC15 | na | 863 | 0.566 | 0.4838 | Yes |
| 31 | MCUB | na | 883 | 0.561 | 0.4956 | Yes |
| 32 | NDUFB5 | na | 902 | 0.554 | 0.5073 | Yes |
| 33 | NDUFV3 | na | 988 | 0.527 | 0.5127 | Yes |
| 34 | NDUFB4 | na | 1015 | 0.517 | 0.5228 | Yes |
| 35 | NDUFA12 | na | 1023 | 0.513 | 0.5345 | Yes |
| 36 | CHCHD3 | na | 1059 | 0.500 | 0.5435 | Yes |
| 37 | PPIF | na | 1060 | 0.500 | 0.5554 | Yes |
| 38 | TIMM10 | na | 1066 | 0.498 | 0.5668 | Yes |
| 39 | NDUFA6 | na | 1079 | 0.492 | 0.5776 | Yes |
| 40 | NDUFA8 | na | 1080 | 0.492 | 0.5893 | Yes |
| 41 | UQCRQ | na | 1103 | 0.484 | 0.5990 | Yes |
| 42 | NDUFS2 | na | 1124 | 0.477 | 0.6087 | Yes |
| 43 | COX4I1 | na | 1125 | 0.476 | 0.6200 | Yes |
| 44 | SDHC | na | 1135 | 0.473 | 0.6305 | Yes |
| 45 | MTX2 | na | 1196 | 0.457 | 0.6364 | Yes |
| 46 | NDUFB10 | na | 1215 | 0.450 | 0.6456 | Yes |
| 47 | UQCRFS1 | na | 1263 | 0.437 | 0.6521 | Yes |
| 48 | ATP5PB | na | 1267 | 0.436 | 0.6622 | Yes |
| 49 | NDUFV1 | na | 1307 | 0.428 | 0.6691 | Yes |
| 50 | NDUFB8 | na | 1330 | 0.423 | 0.6774 | Yes |
| 51 | NDUFS7 | na | 1371 | 0.412 | 0.6838 | Yes |
| 52 | NDUFV2 | na | 1395 | 0.408 | 0.6916 | Yes |
| 53 | TIMM17B | na | 1436 | 0.396 | 0.6977 | Yes |
| 54 | TIMM50 | na | 1455 | 0.393 | 0.7056 | Yes |
| 55 | TIMM22 | na | 1682 | 0.337 | 0.6946 | No |
| 56 | UQCRC2 | na | 1700 | 0.332 | 0.7011 | No |
| 57 | NDUFB9 | na | 1842 | 0.299 | 0.6964 | No |
| 58 | CHCHD6 | na | 1990 | 0.273 | 0.6906 | No |
| 59 | NDUFB1 | na | 1995 | 0.272 | 0.6967 | No |
| 60 | IMMT | na | 2013 | 0.270 | 0.7017 | No |
| 61 | SAMM50 | na | 2097 | 0.254 | 0.7008 | No |
| 62 | BCS1L | na | 2225 | 0.232 | 0.6957 | No |
| 63 | TIMM21 | na | 2269 | 0.226 | 0.6974 | No |
| 64 | UQCRC1 | na | 2326 | 0.217 | 0.6979 | No |
| 65 | GRPEL1 | na | 2491 | 0.193 | 0.6888 | No |
| 66 | NDUFA4 | na | 2726 | 0.160 | 0.6730 | No |
| 67 | NDUFA9 | na | 2879 | 0.141 | 0.6636 | No |
| 68 | PHB | na | 3009 | 0.125 | 0.6557 | No |
| 69 | PHB2 | na | 3057 | 0.119 | 0.6546 | No |
| 70 | APOO | na | 3061 | 0.119 | 0.6572 | No |
| 71 | MICU1 | na | 3262 | 0.092 | 0.6426 | No |
| 72 | NDUFA10 | na | 3412 | 0.073 | 0.6318 | No |
| 73 | SDHA | na | 3488 | 0.064 | 0.6271 | No |
| 74 | MICU3 | na | 3510 | 0.062 | 0.6268 | No |
| 75 | HSPA9 | na | 3575 | 0.055 | 0.6227 | No |
| 76 | DNAJC11 | na | 3589 | 0.054 | 0.6229 | No |
| 77 | NDUFS1 | na | 3763 | 0.033 | 0.6092 | No |
| 78 | ROMO1 | na | 3798 | 0.028 | 0.6070 | No |
| 79 | NDUFAF1 | na | 3921 | 0.014 | 0.5971 | No |
| 80 | IMMP1L | na | 3959 | 0.009 | 0.5942 | No |
| 81 | NDUFS4 | na | 4402 | -0.040 | 0.5581 | No |
| 82 | MCU | na | 4548 | -0.056 | 0.5472 | No |
| 83 | AGK | na | 4571 | -0.058 | 0.5468 | No |
| 84 | TIMM23B | na | 4647 | -0.065 | 0.5420 | No |
| 85 | COX7C | na | 4881 | -0.090 | 0.5247 | No |
| 86 | AFG3L2 | na | 5510 | -0.150 | 0.4755 | No |
| 87 | SLC25A6 | na | 6201 | -0.225 | 0.4230 | No |
| 88 | FOXRED1 | na | 6248 | -0.229 | 0.4246 | No |
| 89 | IMMP2L | na | 6710 | -0.275 | 0.3925 | No |
| 90 | GRPEL2 | na | 7548 | -0.366 | 0.3310 | No |
| 91 | MICU2 | na | 9095 | -0.564 | 0.2147 | No |
| 92 | MTX3 | na | 9355 | -0.607 | 0.2074 | No |
| 93 | APOOL | na | 9563 | -0.640 | 0.2053 | No |
Table: GSEA details [plain text format]

  

Fig 2: GOCC\_INNER\_MITOCHONDRIAL\_MEMBRANE\_PROTEIN\_COMPLEX      
 Blue-Pink O' Gram in the Space of the Analyzed GeneSet

  

Fig 3: GOCC\_INNER\_MITOCHONDRIAL\_MEMBRANE\_PROTEIN\_COMPLEX: Random ES distribution      
 Gene set null distribution of ES for **GOCC\_INNER\_MITOCHONDRIAL\_MEMBRANE\_PROTEIN\_COMPLEX**
